# The digestive systems of carnivorous plants

**DOI:** 10.1093/plphys/kiac232

**Published:** 2022-05-23

**Authors:** Matthias Freund, Dorothea Graus, Andreas Fleischmann, Kadeem J Gilbert, Qianshi Lin, Tanya Renner, Christian Stigloher, Victor A Albert, Rainer Hedrich, Kenji Fukushima

**Affiliations:** Institute for Molecular Plant Physiology and Biophysics, University of Würzburg, Würzburg, Germany; Institute for Molecular Plant Physiology and Biophysics, University of Würzburg, Würzburg, Germany; Botanische Staatssammlung München and GeoBio-Center LMU, Ludwig-Maximilians-University Munich, Munich, Germany; Department of Plant Biology & W.K. Kellogg Biological Station, Michigan State University, Hickory Corners, Michigan 49060, USA; Department of Botany, University of British Columbia, Vancouver, BC V6T 1Z4, Canada; Department of Entomology, The Pennsylvania State University, University Park, Pennsylvania 16802, USA; Imaging Core Facility of the Biocenter, University of Würzburg, Würzburg, Germany; Department of Biological Sciences, University at Buffalo, Buffalo, New York 14260, USA; Institute for Molecular Plant Physiology and Biophysics, University of Würzburg, Würzburg, Germany; Institute for Molecular Plant Physiology and Biophysics, University of Würzburg, Würzburg, Germany

## Abstract

To survive in the nutrient-poor habitats, carnivorous plants capture small organisms comprising complex substances not suitable for immediate reuse. The traps of carnivorous plants, which are analogous to the digestive systems of animals, are equipped with mechanisms for the breakdown and absorption of nutrients. Such capabilities have been acquired convergently over the past tens of millions of years in multiple angiosperm lineages by modifying plant-specific organs including leaves. The epidermis of carnivorous trap leaves bears groups of specialized cells called glands, which acquire substances from their prey via digestion and absorption. The digestive glands of carnivorous plants secrete mucilage, pitcher fluids, acids, and proteins, including digestive enzymes. The same (or morphologically distinct) glands then absorb the released compounds via various membrane transport proteins or endocytosis. Thus, these glands function in a manner similar to animal cells that are physiologically important in the digestive system, such as the parietal cells of the stomach and intestinal epithelial cells. Yet, carnivorous plants are equipped with strategies that deal with or incorporate plant-specific features, such as cell walls, epidermal cuticles, and phytohormones. In this review, we provide a systematic perspective on the digestive and absorptive capacity of convergently evolved carnivorous plants, with an emphasis on the forms and functions of glands.

## Teaser

A comparison of the forms and functions of digestive and absorptive glands in carnivorous plants sheds light on their convergent evolution.

## The carnivorous plant leaf as an all-in-one organ analogous to the animal digestive tract

**Figure 1:**
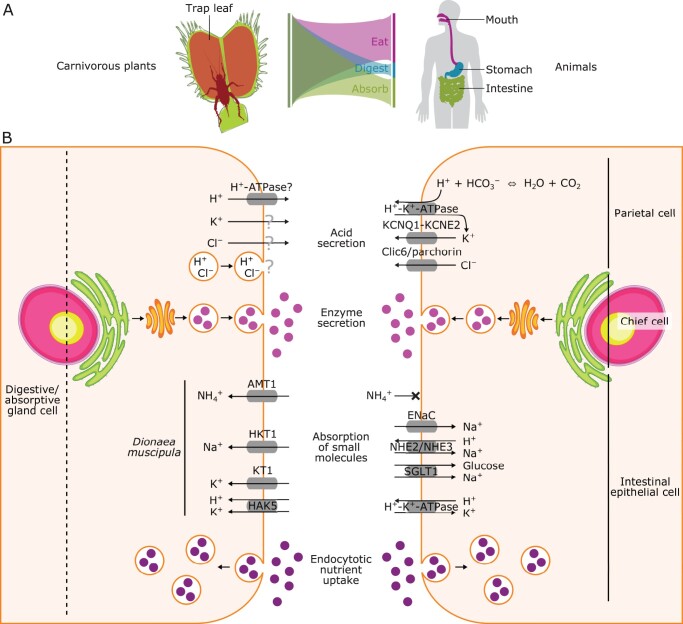
Functional similarities between a trap leaf and a digestive tract. A, The spatial differentiation of the digestive system. The sites for eating, digestion, and absorption are spatially separated in the animal system (symbolized by colors), but not in carnivorous plants (overlapping colors). B, Secretory and absorptive pathways that are discussed in the main text and Box 2. Note that the figure shows an imaginary synthetic cell because interspecies and gland-type-specific differences in these processes are often unknown in carnivorous plants. Among the many secretory and absorptive pathways and membrane proteins identified in parietal cells (Yao and Forte, 2003; Engevik et al., 2020), chief cells (Hirschowitz, 1967), and intestinal epithelial cells (Pácha, 2000; Rajendran et al., 2018; Engevik and Engevik, 2021) in animals, only the counterparts of those characterized in carnivorous plants are shown. The cell wall and cuticle are not shown. The organelles are not shown to scale.

**Figure 2:**
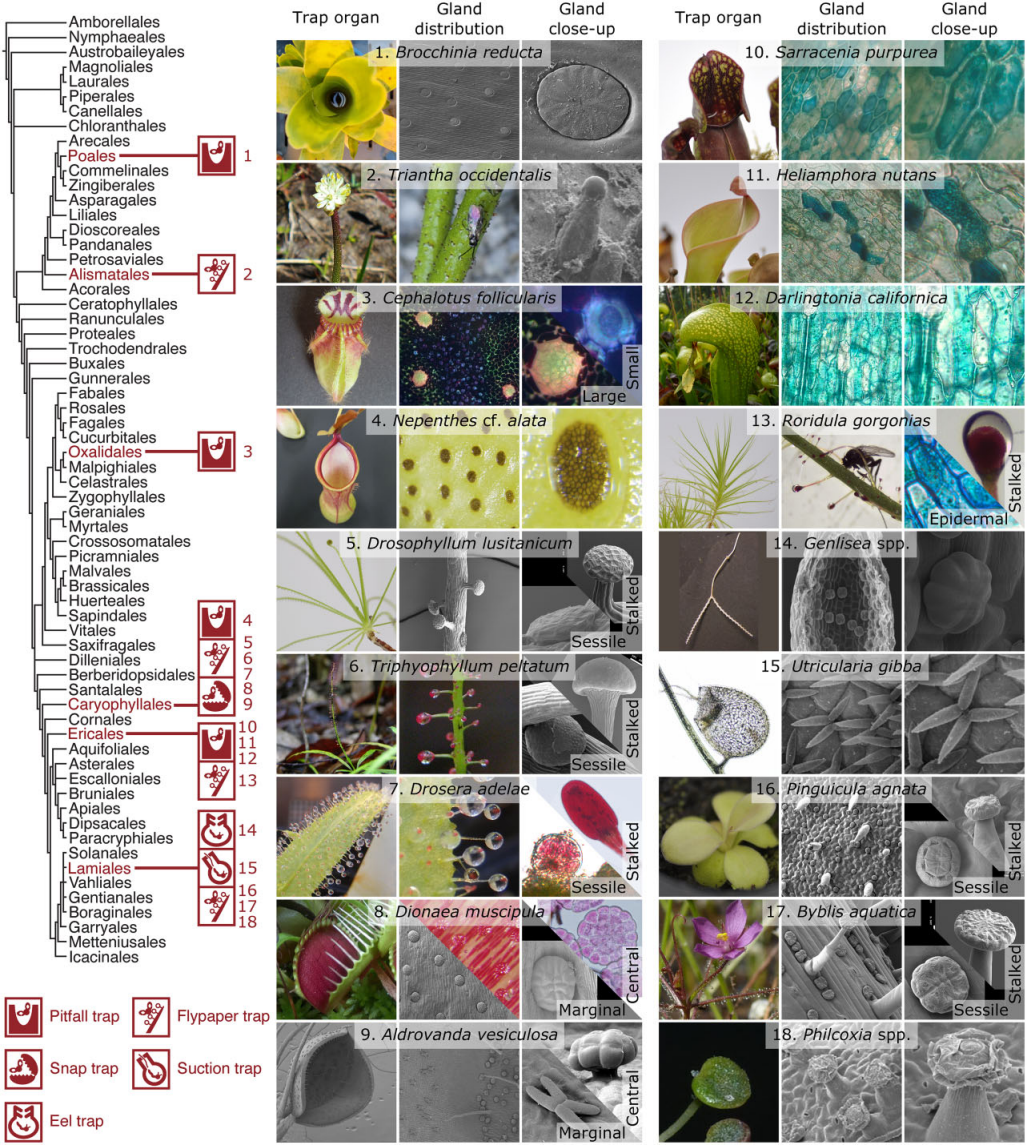
Evolution of glandular cells in carnivorous plants. The order-level phylogeny of flowering plants (The Angiosperm Phylogeny Group et al., 2016) is shown on the left, with lineages containing carnivorous plants and their trap types highlighted in red. Branch lengths have no information. Trap leaves and glands of representative species are shown on the right (for scanning electron microscopy, see Supplemental Methods S1). To increase visibility, methylene blue staining was applied to the glands of *Cephalotus*, *Sarracenia*, *Heliamphora*, *Darlingtonia*, and *Roridula* (Supplemental Methods S2). Whole or parts of the photographs of *Utricularia* and *Philcoxia* were reproduced from the literature (Yang et al., 2009; Pereira et al., 2012). The photographs of *Aldrovanda* were provided by Dirk Becker. Original pictures (including scale bars for microscopic pictures) are available in figshare (https://doi.org/10.6084/m9.figshare.18271529) under CC BY 4.0 (https://creativecommons.org/licenses/by/4.0/).

Like an animal’s mouth, carnivorous plants use their trapping structures to “eat” their prey, primarily small arthropods. All carnivorous plants discovered to date capture their prey using modified leaves called “trap leaves,” except for *Triantha* (false asphodel), which was recently shown to produce flypaper-type traps exclusively on its flower stalks (Lin et al., 2021). Although trap leaves share many functions with animal digestive tracts, there are striking differences in their spatial arrangements (Figure 1). Most vertebrate digestive tracts are divided into functionally specialized organs such as the mouth, stomach, and intestines, where food is digested and absorbed in distinct compartments (Hedrich, 2015). In carnivorous plants, however, the prey does not travel through a digestive tract but instead remains in the same organ where it was captured for subsequent digestion and absorption (comparable to some animals with a blind-ended digestive tract, such as polyps; Steinmetz, 2019). Therefore, in principle, trap leaves are all-in-one organs with multifaceted functions, regardless of trap type (Figure 2). However, in certain trap types, a spatial split of functions may be observed within the organ (i.e. within a single leaf). A striking example is the eel traps of *Genlisea* (corkscrew plants), in which bifurcating arm-like trapping organs are well separated from the digestive chamber (Figure 2).

Most carnivorous plants employ their leaf-derived traps (or parts of these structures) for both photosynthesis and prey capture, while a few plants develop specialized trap leaves in addition to conventional foliar leaves (*Cephalotus* [Albany pitcher plant], *Genlisea*, and some *Utricularia* [bladderworts] species) or compensate for the reduced photosynthetic function of the traps by generating modified shoots (most *Utricularia* species; Fleischmann, 2018; Fleischmann et al., 2018b).

**Figure 3:**
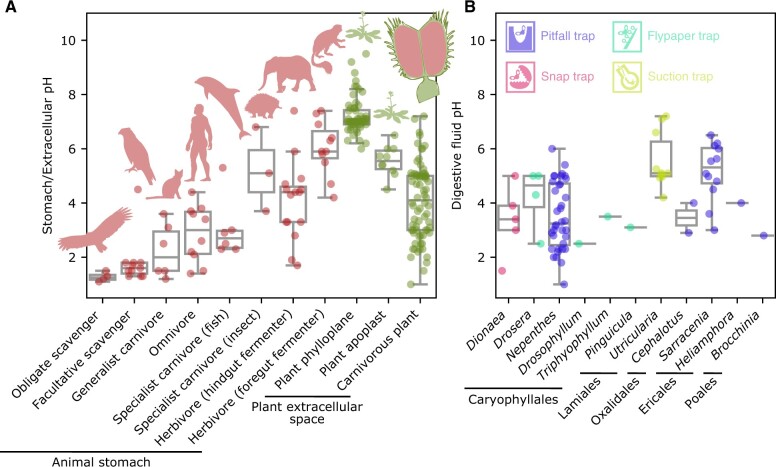
Digestive fluid acidity across the tree of life. A, Extracellular pH in the digestive organs of plants and animals. The plant apoplast and phylloplane (i.e. leaf surface) were included for comparison with the digestive fluid of carnivorous plants. The datasets for animal stomachs and plant phylloplane were obtained from the literature (Beasley et al., 2015; Gilbert and Renner, 2021). The source data for the others are available in Supplemental Table S1. When pH was measured at multiple time points or under multiple conditions, only the lowest value was included. The silhouettes of representative organisms were obtained from PhyloPic (http://phylopic.org). The silhouette of *Cathartes aura* is licensed under CC BY-SA 3.0 (https://creativecommons.org/licenses/by-sa/3.0/) by Sevcik et al. B, pH of the digestive fluids of different carnivorous plant genera. Boxplot elements are defined as follows: center line, median; box limits, upper and lower quartiles; whiskers, 1.5 × interquartile range.

The primary function of the animal stomach is the chemical breakdown of food. The parietal cells of the human stomach secrete hydrochloric acid (Engevik et al., 2020), which creates a highly acidic environment with a pH of approximately 1.5 (Dressman et al., 1990; Russell et al., 1993). The acidic conditions serve as a barrier against food-borne pathogens and provide the optimal environment for digestive enzyme activity (Smith, 2003; Martinsen et al., 2005). Although typically not as acidic as the human stomach, the digestive fluids of carnivorous plants can be highly acidic, often reaching pH 2–3, which is more acidic on average than the gastric acids of insect-eating animals (Beasley et al., 2015; Figure 3;Supplemental Table S1). Akin to the animal stomach, this acidic environment is primarily generated by inorganic acids, mainly hydrochloric acid (Rea, 1982). The molecular machinery that generates hydrochloric acid is largely unknown in many carnivorous plants, but in *Dionaea* (Venus flytrap), active exocytosis coincides with the secretion of calcium, protons, and chloride, suggesting the involvement of vesicle-mediated transport that prevents disturbance of the membrane potentials of gland cells (Scherzer et al., 2017). Alternatively, membrane proteins such as ion channels may be involved in this process, as shown in animals (Figure 1B).

One major proteolytic enzyme activated under acidic conditions in the human stomach is pepsin (Fruton, 2002). Since pepsin contains two aspartic acid residues in its active site, this enzyme belongs to the aspartic protease protein family. Carnivorous plants use enzymes similar to animal pepsin to breakdown animal proteins, as discovered by Charles Darwin (Darwin, 1875). More recently, carnivory-active proteolytic enzymes were isolated from *Nepenthes* (tropical pitcher plants), *Cephalotus*, and *Sarracenia* (North American pitcher plants) and were found to be aspartic proteases (Athauda et al., 2004; Hatano and Hamada, 2008; Rottloff et al., 2016; Fukushima et al., 2017). Although *Dionaea* also secretes aspartic proteases (Schulze et al., 2012; Paszota et al., 2014), cysteine proteases are likely the most abundant proteolytic enzymes in its digestive fluid (Takahashi et al., 2011; Libiaková et al., 2014). Many carnivorous plants possess several additional enzyme classes that degrade various high-molecular weight compounds found in an insect’s body. Examples include chitinases, which breakdown chitin, a component of the arthropod exoskeleton; ribonucleases, which breakdown nucleic acids; and other enzymes, such as amylases, esterases, and phosphatases (Ravee et al., 2018). This rich enzymatic repertoire parallels that of animal digestive systems (Lemaitre and Miguel-Aliaga, 2013; Janiak, 2016). Their evolutionary origin is often linked to defense mechanisms (discussed later), but some enzymes appear to have been coopted from other ancestral functions (Kocáb et al., 2020). The secretion of proteins such as digestive enzymes is assumed to occur via the conventional secretory pathway common to plants and animals (Wang et al., 2018), although other pathways may also be involved (see Supplemental Text S1). However, in several carnivorous plants, prey digestion is partly or fully performed by associated microorganisms that live in the trap—comparable to the intestinal microbiota in animals, which are also essential for digestion (Hanning and Diaz-Sanchez, 2015).

Digested food in the human stomach is transported to the intestine, where degraded products are absorbed. Numerous transporter proteins in animal intestines participate in the uptake of a variety of nutrients such as ions, sugars, amino acids, and peptides (Pácha, 2000; Jackson and Mclaughlin, 2006; Bröer, 2008; Boudry et al., 2010; Schmidt et al., 2010; Estudante et al., 2013; Bröer and Fairweather, 2018; Rajendran et al., 2018; Engevik and Engevik, 2021). Several transporter proteins involved in nutrient absorption have been identified in *Dionaea*, whose repertoire may be distinct from its human counterparts (Figure 1B). Although transporters usually absorb only specific compounds, mammalian intestines, often during early postnatal life, can encapsulate extracellular macromolecules in vesicles and absorb them into cells intact (Pácha, 2000). This process, endocytosis, enables relatively nonselective nutrient uptake. This combination of membrane protein action and endocytosis is also found in carnivorous plant leaves (Adlassnig et al., 2012). Thanks to their variety of digestive enzymes and absorption pathways, carnivorous plants can utilize a wide range of prey-derived small and large molecules; the latter include proteins, nucleic acids, chitins, and glucans (Matušíková et al., 2018).

## Digestive and absorptive glands

Glands are not unique to carnivorous plants, as many vascular plants possess glands for secreting various materials, including nectar, mucilage, resin, salts, aromatic compounds, and physiological residues (Callow et al., 2000; Mehltreter et al., 2021). Such exudates often contain hydrolytic enzymes and other proteins (Shepherd and Wagner, 2007; Heil, 2011). Some of the most commonly secreted proteins are pathogenesis-related proteins, which prevent fungal and bacterial growth via hydrolytic activity or function in processes such as lipid transfer and defense signaling (Sels et al., 2008). As such, the glandular functions in trap leaves may be considered convergent exaptations of the various repertoires of structures and exudates found across angiosperm phylogeny (Juniper et al., 1989; Fleischmann et al., 2018a, 2018b). For example, in a study of 19 noncarnivorous plants, 15 species were found to have protease activity in their glandular trichome secretions (Spomer, 1999).

Like other secretory tissues, such as hydathodes, salt glands, and nectaries (Fahn, 1988; Vogel, 1998), the glands of carnivorous plants are distinguished by their physiological functions, which are related to prey digestion and nutrient absorption. Their morphology is often well differentiated from that of other epidermal cells (Juniper et al., 1989), but in Sarraceniaceae, epidermal cells that may differ only slightly in size from surrounding cells exhibit cuticular permeability and endocytotic activity, the hallmark features of carnivorous plant glands (Koller-Peroutka et al., 2019). Digestive glands secrete mucilage, ions, and proteins including digestive enzymes (Darwin, 1875; Juniper et al., 1989). The same or morphologically distinct glands then absorb the degraded compounds via the activities of membrane transport proteins and endocytosis. The occurrence of more than one type of gland is common in carnivorous plant groups (Juniper et al., 1989), but their functional differentiation is not clearly understood in many species. Although glands are defined based on their secretory or absorptive functions, they are often judged to be glands based on their morphology and localization. As such, it has been assumed that these morphological differences come with functional differences in terms of digestive and absorptive capabilities, but more recent evidence points toward at least partial overlap in functions between different types of glands in different lineages. For example, phosphatase activity could be detected in both sessile and stalked glands of *Pinguicula* (butterworts; Płachno et al., 2006), suggesting both glands are capable of digestion. There is also evidence of endocytotic uptake in both types of glands of *Drosophyllum* (Adlassnig et al., 2012). However, a more comprehensive study comparing all relevant genera and glands will be necessary to dispel the initial dogma completely.

## Evolution of different trap types from flypaper traps

The flypaper trap is the most frequently occurring type of trap in carnivorous plants, having independently evolved in at least six lineages, including three in the Lamiales alone (in *Pinguicula*, *Byblis* [rainbow plants], and *Philcoxia*; Schäferhoff et al., 2010; Fleischmann et al., 2018a, 2018b), at least one each within the Caryophyllales and Ericales (Albert et al., 1992), as well as the recently discovered carnivorous inflorescences of *Triantha occidentalis* (Lin et al., 2021; Figure 2). Some plants are considered “para-carnivorous,” that is, sticky plants that casually trap insects but do not make use of the trapped “prey,” for example, *Ibicella* (Płachno et al., 2009) and *Stylidium* (Darnowski et al., 2006). Note that the features required for the carnivorous syndrome are controversial and vary among researchers (Adamec et al., 2021); the term “para-carnivorous” is not clear-cut and does not imply a “transitional species” on the way to becoming a carnivorous plant. In any case, even more disparate species throughout the angiosperm phylogeny possess sticky trichomes (likely upward of thousands of species), including ones that are unequivocally not currently considered carnivorous or para-carnivorous; instead, they entrap arthropods primarily for herbivore defense, as exemplified by several Lamiales and Solanaceae species (Adlassnig et al., 2010; Bar and Shtein, 2019; Adamec et al., 2021; Chase and Christenhusz, 2021).

Flypaper traps may have given rise to all other trap types (Albert et al., 1992; Fleischmann et al., 2018b). In the carnivorous Caryophyllales, the most parsimonious hypothesis is that the flypaper trap type is plesiomorphic, with snap traps and pitfall traps derived from ancestors with sticky traps (Heubl et al., 2006; Renner and Specht, 2011; Fleischmann et al., 2018a, 2018b). Similarly, the flypaper trap of *Pinguicula* is sister to the two other trap types in Lentibulariaceae in carnivorous Lamiales (Müller et al., 2006). Although possibly not an immediate phylogenetic sister (Löfstrand and Schönenberger, 2015), the pitfall traps in Ericales are also closely related to those of a flypaper trap lineage (*Roridula*).

Evidence suggests that mucilage production in ancestral flypaper traps has been retained in some of these other trap types. For instance, both *Utricularia* and *Genlisea* (suction and eel traps, respectively; Lentibulariaceae) produce bifid trichomes with mucilage secretions on their traps and globose glands that secrete mucilage on their leaves (Taylor, 1989; Płachno et al., 2006; Adlassnig et al., 2010; Fleischmann, 2012). Interestingly, certain species of the pitfall-trapping *Nepenthes* genus produce a mucilage-derived, highly viscoelastic digestive fluid (Gaume and Forterre, 2007; Bauer et al., 2011; Bonhomme et al., 2011; Renner and Specht, 2011) that aids in prey retention (Di Giusto et al., 2008; Moran et al., 2013; Bazile et al., 2015; Gaume et al., 2019; Kang et al., 2021), representing a type of hybrid trapping strategy reminiscent of their close relatives *Drosera* (sundews). Exploring mucilage-mediated interactions with other organisms could shed light on the evolution of carnivorous plants (Box 1).

## Mucilage production and secretion mechanisms

Little is known about the production and secretion of mucilage across the various carnivorous plant lineages, although limited evidence is available for members of the Caryophyllales (Droseraceae and Drosophyllaceae) and Lamiales (Lentibulariaceae). The mucilage of *Drosera binata* contains an acidic polysaccharide comprising arabinose, galactose, glucuronic acid, mannose, and xylose (Gowda et al., 1982; Erni et al., 2008), while the acidic polysaccharide of *D. capensis* is slightly modified and is composed of ester sulfate, galactose, glucuronic acid, mannose, and xylose (Rost and Schauer, 1977). Across *Drosera*, however, the Golgi apparatus appears to be responsible for both mucilage production and secretion (Schnepf, 1961a; Dexheimer, 1978; Outenreath and Dauwalder, 1986; Lichtscheidl et al., 2021). The glands of the Caryophyllales carnivores *Triphyophyllum* and *Drosophyllum* also produce acidic secretions. The constituents of these secretions in *Triphyophyllum* are unknown, but those in *Drosophyllum* contain carbohydrates produced by the Golgi apparatus (Schnepf, 1961b, 1963a, 1972; Marburger, 1979). Interestingly, the polysaccharide found in *Drosophyllum* mucilage differs from that of *Drosera* and includes the monomers arabinose, galactose, glucuronic acid, rhamnose, and xylose, as well as ascorbic acid (Schnepf, 1963b). Similarly, in *Pinguicula* of the Lentibulariaceae, polysaccharides are prevalent in the sticky mucilage and are likely transported intracellularly by vesicles derived from the Golgi apparatus, as observed in *Drosera* and *Drosophyllum* (Heslop-Harrison and Knox, 1971; Vassilyev and Muravnik, 1988). In *Pinguiculavulgaris*, the mucilage itself is stored within vacuoles, as well as the periplasmic space, before being released to the gland surface (Vassilyev and Muravnik, 1988; Adlassnig et al., 2010). In closely related *Genlisea*, mucilage is also stored in the periplasmic space of secretory glands (Płachno, 2008). A notable exception to the polysaccharide-rich mucilages of carnivorous plants is the genus *Roridula*, which secretes resinous compounds and will be discussed further below.

## Convergent co-option of digestive enzymes

The highly repeated convergent evolution of plant carnivory (Figure 2) suggests that the transition from the noncarnivorous to carnivorous state was broadly genetically accessible to a wide range of angiosperm lineages. In agreement with this idea, all known digestive enzymes of carnivorous plants are not unique but originated from ubiquitous gene families found throughout flowering plants (Fukushima et al., 2017). In particular, defense-related genes tend to be repurposed for digestive physiology (Bemm et al., 2016), with possible changes in biochemical properties occurring through positively selected convergent amino acid substitutions (Fukushima et al., 2017). Several proteins involved in plant defense, including hydrolytic enzymes, are secreted to the extracellular space (Lee et al., 2004). Pathogenic microbes, fungi, and both phytoparasitic and herbivorous (and sometimes prey) insects share many biological components (e.g. chitin), perhaps providing a ready basis for the evolutionary co-option of enzyme-encoding genes.

## Secretion of digestive enzymes

Various digestive enzymes have been identified in the digestive fluid of carnivorous plants and are thought to be secreted from glands (Heslop-Harrison, 1975; Juniper et al., 1989; Ravee et al., 2018; Hedrich and Fukushima, 2021). In particular, extracellular phosphatase activity is a widely detected, key characteristic of the glands of carnivorous plants (Płachno et al., 2006, 2009; Lin et al., 2021). However, thus far, genes encoding secreted phosphatases have only been isolated in *Nepenthes* and *Cephalotus* (Fukushima et al., 2017). Additionally, commonly used dye-based method appears to label both intracellular and extracellular phosphatase activity following intensive endocytosis (Płachno et al., 2006), which may confound the extracellular signal with the intracellular noise of housekeeping phosphatases. Not much is known about the tissue-specific secretion and localization of digestive enzymes, except for the phosphatases and the aspartic protease Nepenthesin I expressed in the parenchyma around the glands of *Nepenthes* (Athauda et al., 2004). In *Cephalotus*, which conditionally produces distinct trapping leaves (Fukushima et al., 2017, 2021), approximately half of the genes encoding digestive fluid proteins are specifically expressed in pitcher leaves, but the other half are also expressed in the photosynthetic, nontrapping leaves (Fukushima et al., 2017). Trap-preferential gene expression has been reported in other species as well, with a few exceptions (Rottloff et al., 2011, 2013; Nishimura et al., 2013; Arai et al., 2021). Perhaps, these digestive enzymes exist in a bifunctional state for defense and digestion, or perhaps they are encoded by sub-/neofunctionalized duplicates specialized for digestive physiology, which might influence the tissues and cell types that secrete the enzymes.

## Proton transport

The acidity of digestive fluid is a hallmark of carnivorous plants. Although the pH varies among carnivorous plant genera (Figure 3B), the digestive fluids of carnivorous plants are often more acidic than the gastric juices of animals with specialized feeding habits, including insect-eating carnivores (Figure 3A). This strong acidity has several potential benefits, including the capacity for (1) killing prey (Bazile et al., 2015); (2) suppressing microbial growth (Buch et al., 2013); (3) acid-mediated auto-activation of aspartic proteases, a process similar to pepsin activation in the animal stomach (Runeberg-Roos et al., 1991; Fruton, 2002; Buch et al., 2015); (4) efficient degradation of proteins and other substrates by digestive enzymes with acidic pH optima (An et al., 2001; Saganová et al., 2018); and (5) nutrient absorption driven by proton gradients. Protons and potassium ions are thought to be the primary cations in some carnivorous plant species due to their abundance and the scarcity of other cations (Nemček et al., 1966; Juniper et al., 1989; Scherzer et al., 2013, 2015; Gao et al., 2015; Fasbender et al., 2017; Box 2). Although the pH of digestive fluid varies among species, its acidity is usually higher than the apoplastic pH in other plants (Figure 3A). Compared to other pitcher plants, many Sarraceniaceae species rely more on microbes than their own digestive enzymes (Luciano and Newell, 2017), likely explaining why the liquid in their pitchers tends to be less acidic than that of other carnivorous plants (Figure 3). In many carnivorous plant groups, the digestive fluid is acidic even in the resting state and becomes more acidic upon prey capture (Supplemental Table S1).

The strong acidity of digestive fluid can be attributed to the activity of proton pumps (Rea, 1984). This view was supported by pharmacological treatment of *Nepenthes* with H^+^-pump inhibitors and an activator that especially affected plasma membrane H^+^-ATPases (An et al., 2001). Noninvasive microelectrode ion flux measurements confirmed that the gland cells in *Nepenthes* and *Dionaea* release protons into the pitcher or snap-trap lumen (Moran et al., 2010; Scherzer et al., 2017). In *Nepenthes*, the putative plasma membrane proton pump gene *NaPHA1* is expressed in glands (An et al., 2001). In *Dionaea*, the levels of vacuolar AHA10-type proton pump transcripts changed in response to coronatine, which mimics bioactive jasmonic acid and induces some prey-capture responses in carnivorous Caryophyllales, a process likely related to acid secretion by exocytotic vesicles (Scherzer et al., 2017; Supplemental Text S1). Future research should address which proton pumps are responsible for fluid acidification and how they differ among carnivorous species.

## Anion transport

To generate hydrochloric acid, both chloride and protons must be excreted into the digestive fluid. Classical pharmacological analyses with metabolic inhibitors demonstrated that the ionic gradients between digestive fluid and gland cells are actively modulated in carnivorous plants (Juniper et al., 1989). Chloride ions are a principal anion in the digestive fluids of some carnivorous species, such as *Nepenthes* spp. (Morrissey, 1955; Nemček et al., 1966). In these pitcher plants, the release of chloride ions coincides with the secretion of proteases (Lüttge, 1966), as in *Dionaea* (Rea et al., 1983; Scherzer et al., 2017) and *Pinguicula* (Heslop-Harrison and Heslop-Harrison, 1980). In *Dionaea*, the vacuolar voltage-dependent ChLoride Channel (CLC) is implicated in chloride transport during prey digestion (Scherzer et al., 2017). Since digestive fluid contains only trace amounts of organic acids (Voelcker, 1849; Morrissey, 1955), it appears that organic anions such as malate (which functions in osmotic regulation in certain plant cells) do not play major roles in this process (Fernie and Martinoia, 2009; Araújo et al., 2011; López-Arredondo et al., 2014). However, organic acids are relatively abundant in the traps of *Utricularia*, even though the fluid pH is close to neutral (Sirová et al., 2011).

## Ammonium absorption

In contrast to the digestive tracts of animals (Romero-Gómez et al., 2009), ammonium likely serves as the preferred form of nitrogen for uptake in carnivorous plants (Figure 1B). After prey capture, ammonium is released into the digestive fluid in *Dionaea* (Scherzer et al., 2013). The addition of pure protein also resulted in ammonium accumulation, and the relative abundance of released amino acids indicates that the enzymatic deamination of glutamine, in particular, produces ammonium in the digestive fluid of *Dionaea* (Scherzer et al., 2013). Tracer experiments supported the notion that nitrogen, likely in the form of ammonium, is separated from the carbon skeleton of glutamate in digestive fluid (Fasbender et al., 2017). In multiple carnivorous plants, ammonium transporters (AMTs) appear to play pivotal roles in ammonium uptake. Transporters for nitrogenous compounds in *Nepenthes* often show negligible expression in glands, except for AMT1 (Schulze et al., 1999). *AMT1* transcripts are localized exclusively to the head cells of the gland, pointing to the involvement of AMT1 in ammonium uptake. Likewise, in *Dionaea*, *AMT1* shows gland-specific expression, with further upregulation following coronatine treatment (Scherzer et al., 2013). *Cephalotus* also has an *AMT1* gene that shows preferential expression in pitcher leaves (Fukushima et al., 2017). Interestingly, some *AMT1* genes in Arabidopsis (*Arabidopsis thaliana*) are highly expressed in roots and are thought to be involved in the uptake of ammonium ions from the soil (Gazzarini et al., 1999; Rawat et al., 1999), suggesting possible co-option of this gene from roots to traps in multiple lineages.

## Membrane trafficking

The direct transport of nutrients via membrane proteins is not the only way substances are absorbed and distributed by cells. Large molecules, such as whole proteins and degraded peptides, can be taken up and released via endocytosis and exocytosis, respectively (Battey et al., 1999; Doherty and McMahon, 2009; Paez Valencia et al., 2016). Active endocytosis is observed in the glands of many carnivorous lineages (Adlassnig et al., 2012). In *Nepenthes*, for example, a few small vesicles were observed within gland cells 1 h after the application of a fluorescent tracer, and by 30 h they combined into one or a few large vesicles that occupied most of the cell volume (Adlassnig et al., 2012).

Membrane trafficking must also be involved in the export of digestive enzymes. Newly synthesized digestive enzymes could follow the classical pathway of protein secretion, in which proteins are synthesized in the endoplasmic reticulum and modified in the Golgi apparatus to be packaged into vesicles in the trans-Golgi network and shuttled out via the plasma membrane (Battey et al., 1999; Cui et al., 2020). Indeed, exosome formation was observed in the glands of coronatine-stimulated *Dionaea* (Hawes et al., 1991; Thiel and Battey, 1998; Scherzer et al., 2017) and other species (Juniper et al., 1989).

## Cuticular permeability

To exchange substances efficiently, the plasma membranes of gland cells must be accessible to the external environment. The plant epidermis is usually protected by a continuous cuticle, but gland cells of carnivorous plants often show cuticular pores or gaps that allow the passage of small molecules. The presence of such cuticular discontinuities has been revealed in many carnivorous plants using electron microscopy and staining with dyes such as methylene blue, which cannot penetrate intact cuticles (Juniper et al., 1989; Płachno et al., 2007; Adlassnig et al., 2012; Koller-Peroutka et al., 2019; Lichtscheidl et al., 2021). While the glands of many species exhibit cuticular permeability, there are some inter-species differences (Adlassnig et al., 2012). In *Drosera*, both stalked and sessile glands show cuticular permeability. *Cephalotus* produces small and large glands, but only small glands show clear cuticular permeability. Dye staining appears to correspond well with functional maturity; in *Dionaea*, immature glands do not stain, and only mature glands show clear permeability. Using fluorescent tracers, endocytotic activity was detected in cells exhibiting cuticular permeability (Adlassnig et al., 2012). In carnivorous Ericales (Sarraceniaceae and Roridulaceae), nutrient uptake is achieved through cuticular pores and an underlying digestive epithelium (Juniper et al., 1989; Anderson, 2005; Płachno et al., 2006) that functions as a gland. The genetics underlying cuticular discontinuity remain unknown.

## Hormonal regulation of gland cell physiology

The digestive systems of carnivorous plants have a likely origin in defense mechanisms against herbivores (Hedrich and Fukushima, 2021). Considering that phytohormones regulate diverse physiological processes, such as plant growth, abiotic stress resistance, and defense against pathogens and insects, it is highly likely that their roles extend to digestive physiology (Pavlovič and Mithöfer, 2019). Jasmonate accumulation during prey capture has been directly observed in *Drosera*, *Aldrovanda* (waterwheel plant), and *Nepenthes* (Nakamura et al., 2013; Yilamujiang et al., 2016; Krausko et al., 2017; Jakšová et al., 2021). In *Dionaea*, jasmonates induce trap closure and digestive fluid secretion (Escalante-Pérez et al., 2011; Libiaková et al., 2014; Pavlovič and Mithöfer, 2019), coupled with proton efflux (Scherzer et al., 2017). While jasmonate induced a carnivory-related response in Caryophyllales species, no effect was detected in *Pinguicula* and *Utricularia* (Kocáb et al., 2020; Jakšová et al., 2021). Although several other phytohormones are also important in plant defense (Berens et al., 2017), the application of abscisic acid, salicylic acid, gibberellin, and indole-3-acetic acid had no detectable effect on the trapping and digestive physiology of *Dionaea*, *Drosera*, or *Pinguicula* (Escalante-Pérez et al., 2011; Libiaková et al., 2014; Krausko et al., 2017; Pavlovič et al., 2017; Kocáb et al., 2020). In contrast, salicylic acid induced trap closure in *Aldrovanda*, although the observed pharmacological damage questions its physiological interpretation (Jakšová et al., 2021). The roles of these and other phytohormones, including ethylene, cytokinins, and brassinosteroids, remain largely unexplored.

## Gland morphology in Oxalidales

Oxalidales has only one carnivorous member, *Cephalotus follicularis* of the monotypic family Cephalotaceae, which remains quite isolated phylogenetically and morphologically in this angiosperm order (Fleischmann et al., 2018b). Although *Cephalotus* uses pitcher-shaped leaves as pitfall traps similar to those of the independently evolved carnivorous lineages *Nepenthes* and Sarraceniaceae, the arrangement and types of glands are lineage specific. Unlike in *Nepenthes*, the lower part of the inner pitcher wall is not evenly endowed with glands in *Cephalotus* (Moran et al., 2010); instead, it has two opposing areas where the glands are densely localized (Figure 2). Within these gland patches, both small and large glands are embedded in the epidermis and are easily distinguished. From a purely visual point of view, small gland cells can be described as immobile stomatal guard cells whose aperture is plugged with a “wall plug” comprising a thickened cell wall (Juniper et al., 1989). Large glands consist of multiple (25–200) cells arranged in a dome-like pattern forming clusters of different sizes (Vogel, 1998; Supplemental Text S2). Large clusters are found in the glandular patch, and the glands gradually become smaller from the pitcher wall up to its peristome (Juniper et al., 1989; Vogel, 1998). The small glands have permeable cuticles (Adlassnig et al., 2012) and various enzyme activities such as esterase, protease, and phosphatase activity (Juniper et al., 1989; Płachno et al., 2006). The large glands have impermeable cuticles (Adlassnig et al., 2012), and only acid phosphatase activity (Płachno et al., 2006) has been demonstrated. These differences gave rise to the idea that *Cephalotus* developed a division of labor in its secretory systems: large glands for fluid production and small glands for digestive enzyme production (Juniper et al., 1989). Whether such a strict division of labor exists or whether these activities overlap remains a question for future research. Analysis of gland morphology pointed to a likely evolutionary connection between somatic guard cells and small glands (Lloyd, 1942) but not large glands (Parkes and Hallam, 1984), although such morphological (dis-)similarity does not provide conclusive evidence for their evolutionary (un)relatedness (Juniper et al., 1989).

## Gland morphology in Caryophyllales

Some of the most well-known carnivorous plants are found in the noncore group of the order Caryophyllales (a.k.a., Nepenthales), ranging from the sundew and Venus flytrap (both Droseraceae) to the pitcher plants of the Nepenthaceae; this order includes genera with a variety of trap types (Supplemental Text S3; Figure 2). Glandular trichomes are prevalent in the lineages sister to the carnivorous group, such as *Plumbago* (Supplemental Text S3), and these trichomes may be homologous to those in caryophyllid carnivores. A carnivorous common ancestor of Caryophyllales might have already developed two types of glands, stalked and sessile (Heubl et al., 2006), although a stochastic character mapping analysis did not necessarily support such a scenario (Renner and Specht, 2011). Only a single type of digestive gland maintains the pitcher fluid of *Nepenthes* by releasing enzymes and absorbing nutrients (An et al., 2001; Adlassnig et al., 2012). A piece of epidermis arches above each digestive gland (Owen, Jr., 1999; Wang et al., 2009). These structures are morphologically similar to the lunate cells of the upper parts of the pitcher, which are thought to provide difficult locomotive terrain for trapped insects (Wang et al., 2009, 2018; Wang and Zhou, 2016). A continuous layer of epidermal cells curves underneath the gland, with vascular cells in close proximity (Owen, Jr., 1999). The stalked glands in the other carnivorous Caryophyllales are vascularized whereas the glands of all other carnivorous plants are nonvascularized (Fenner, 1904; Lloyd, 1942; Juniper et al., 1989; Fleischmann et al., 2018b). In *Drosera*, these glands are called tentacles due to their exceptional anatomical and physiological characteristics. Nitschke (Nitschke, 1861) suggested that these organs represent modified leaf pinnae or outgrowths of the lamina margin, a theory that has since been refuted (Lloyd, 1942). In ∼90% of *Drosera* species (Fleischmann et al., 2018a), increasing numbers of tentacles move toward the captured prey, likely to increase the contact surface area with the prey (Juniper et al., 1989). It was originally believed that the site of mechanosensation was the neck of the stalked cells, directly under the gland head, where the stalk is most bendable (Williams, 1976). However, transcripts of the stretch-activated ion channel gene *FLYCATCHER1* (*FLYC1.1* and *FLYC1.2*) were recently found to be localized specifically to the outer secretory cells of the glandular head, whereas in *Dionaea*, *FLYC1* transcripts were specifically detected in sensory cells (in which most trigger hair flexure occurs; Procko et al., 2021), pointing to the evolutionary connection between digestive glands and Venus flytrap trigger hairs. These trigger hairs invoke rapid trap closure via action potentials, but little is known about the associated channels (Böhm and Scherzer, 2021), except for FLYC1, which functions in mechanosensing (Procko et al., 2021), and the Shaker-type channel K^+^ channel *Dionaea muscipula* 1 (KDM1), which functions in K^+^ re-uptake during the hyperpolarization phase (Iosip et al., 2020). The X-shaped quadrifid digestive glands of the aquatic plant *Aldrovanda* (Droseraceae) show remarkably similar morphology to those of the nonrelated Lamialean genus *Utricularia* (Lentibulariaceae). This gland shape increases the surface area of the expanded gland head cells in plants with an aquatic lifestyle.

## Gland morphology in Lamiales

Among Lamiales, *Byblis* and *Philcoxia* are passive flypaper-type carnivorous plants with relatively few species, whereas Lentibulariaceae is a large family comprising three genera with different trapping mechanisms: *Pinguicula* with flypaper traps, *Genlisea* with eel traps, and *Utricularia* with suction traps (Supplemental Text S4). As in other flypaper-type carnivorous plants, *Byblis*, *Pinguicula*, and at least some species of *Philcoxia* show dimorphism, with stalked and sessile glands (Figure 2). The terminal cells of their glands form head-like structures, except in *Utricularia*, where they develop arm-like elongations, like those in the glands located at the trap margins of *Aldrovanda* (Droseraceae). The type of cuticular discontinuity varies among Lentibulariaceae genera (Płachno et al., 2007). The nonvascularized stalked glands of *Pinguicula* produce mucilage via a unique mechanism among carnivorous plants. It has been suggested for three *Pinguicula* species that during maturation, the gland fills with digestive fluid and undergoes autolysis, leaving dead cells full of mucilage (Heslop-Harrison and Heslop-Harrison, 1981). Thus, *Pinguicula* might be incapable of regenerating the gland after excretion. However, a study of another species provided compelling evidence that the glands remain active during digestion (Vassilyev and Muravnik, 1988). This discrepancy, which may stem from interspecies differences, should be reexamined in the future. In Lentibulariaceae, like in most Lamiales, gland cells are polyploid, which likely aids in their increased physiological activity (Fleischmann et al., 2018b).

## Gland morphology in Ericales

Sarraceniaceae comprises three extant taxa: *Heliamphora* (sun pitchers), *Darlingtonia* (cobra lily), and *Sarracenia*. Their pitfall traps share an elongated, funnel-shaped silhouette that in some species collects rainwater, while in other species, an enlarged pitcher lid prevents the pitchers from being flooded (Chen et al., 2018). In all of these pitcher plants, the prey falls into the pitcher, where it is then digested. For glands, Sarraceniaceae utilize morphologically unremarkable epidermal cells called digestive epithelia (Figure 2), wherein endocytosis occurs (Koller-Peroutka et al., 2019). Dye staining of digestive zones revealed regions of these epidermal cells with permeable cuticles (Koller-Peroutka et al., 2019).

Ericales contains an additional carnivorous genus, *Roridula*, with flypaper-type traps. In addition to digestive epithelia, it has morphologically distinctive glands that sit on top of a multicellular trichome. Each globular gland contains an indentation at its pole for increased surface area (Figure 2). The longest trichomes are thought to be responsible for prey entanglement, the shortest ones for immobilization, and the medium-sized ones for slowing down prey movements (Voigt et al., 2009). The adhesive power of the glue, which in *Roridula* is resinous (in all other sticky carnivorous plants, it is aqueous), is derived from triterpenoid compounds (Simoneit et al., 2008), making it a lipophilic resin that is sticky even underwater (Voigt et al., 2015). Due to this lipophilic secretory nature, *Roridula* exhibits unique features, such as digestive mutualism with symbiotic hemipterans (Ellis and Midgley, 1996) and a lack of digestive enzymes in the fluid (Lloyd, 1934) (Box 1; Supplemental Text S5). However, even in the absence of symbionts, *Roridula* seems to be capable of nutrient uptake from prey to some extent (Płachno et al., 2009). Digestive epithelia seem to be the likely site of nutrient uptake, since phosphatase activity was only found in the epidermis of the leaves, rather than stalked glands (Płachno et al., 2009).

## Gland morphology in Poales

Many epiphytic bromeliads collect water in a “tank” formed by tightly arranged rosette leaves (Ladino et al., 2019). Insects and other organic material can accumulate in these small bodies of water, termed phytotelmata. Among bromeliads, *Brocchinia reducta, B. hechtioides*, and *Catopsis berteroniana* are recognized as carnivorous (Fish, 1976; Frank and O’Meara, 1984; Givnish et al., 1984, 1997; Fleischmann et al., 2018b). *Brocchinia reducta* actively utilizes dead matter by absorbing free nutrients, earning the species a spot among carnivorous plants (Givnish et al., 1984; Benzing et al., 1985). *Brocchinia hechtioides* is less well studied, but it shares many carnivory-associated traits with *B. reducta*, such as overall morphology and habit, acidic tank water, emission of nectar-like scent, presence of insect carcasses in the tank and similar trichome structure (Givnish et al., 1997). The glandular trichomes of *B. reducta* have very weak phosphatase activity, but it remains unclear if they produce digestive enzymes themselves (Płachno et al., 2006): digestion is likely handled by bacteria and inquilines (Leroy et al., 2016). In *B. reducta*, glands are scattered across the entire leaf surface instead of being restricted to specific zones as in other pitcher plants (Juniper et al., 1989). These glandular trichomes are embedded in epidermal cavities, with the heads even with the inner tank surface (Benzing et al., 1985). The gland cap is radially organized, but it lacks the central disc cells typically observed in Tillandsioideae species such as *Catopsis* (Benzing et al., 1985). In that genus, four central disc cells are surrounded by multiple layers of cells, with each layer increasing in cell number (Benzing, 1976).

*Paepalanthus bromelioides* belongs to the Eriocaulaceae and even though not directly related to the bromeliads, its habitus is very similar to them: A rosette of leaves forms a water tank, the leaves are covered in wax possibly slippery to insects and produce UV-reflecting powder (Figueira et al., 1994). Although its carnivorous nature is under debate among scientists (Fleischmann et al., 2018b), some evidence points toward the plant being able to partially utilize nitrogen from insect carcasses and feces of inquiline predators falling into the water tank (Nishi et al., 2013). This species may be considered carnivorous under the confines of digestive mutualism but remains severely understudied. While there are mentions of hydrophilous trichomes near the leaf bases, a detailed description of any digestive glandular structure has yet to be provided (Figueira et al., 1994).

## Gland morphology in Alismatales

*Triantha* is the only carnivorous lineage in the monocot order Alismatales. Carnivory has only been demonstrated in *T.occidentalis* (Lin et al., 2021), but it may also exist in the three other species of the genus. *Triantha* is unique among carnivorous plants in that it captures prey (small insects) solely on its sticky flowering stems and thus only during the flowering season, perhaps to enhance reproductive fitness (Lin et al., 2021). Unlike other genera in Tofieldiaceae, *Triantha* contains glandular hairs along its inflorescences (Packer, 2003), with fewer, smaller glands along the lower part of the stem, which is less sticky. The cylindrical glands are multicellular and typically concave at the top. The internal structure of the gland remains to be studied. The flowering stem of *T. occidentalis* secretes phosphatase (Lin et al., 2021), and phosphatase substrate hydrolysis is strongest on the glands, which appear to specifically secrete this digestive enzyme. The other digestive enzymes that *Triantha* may produce and the mechanism by which the plant absorbs nutrients remain to be demonstrated.

## Concluding remarks

Studies of multiple carnivorous plant lineages revealed that various properties of glands have been acquired in parallel, such as gland dimorphism, cuticular permeability, acid secretion, endocytotic activity, and digestive enzyme secretion. However, the underlying molecular mechanisms are often unknown; thus, it is not clear whether these similar traits are brought about by the functions of common genes (see “Outstanding Questions”). The exception is the genes encoding digestive enzymes, in which multiple cases of convergent co-options are well documented. In contrast, the actions of phytohormones and gland morphology tend to be lineage specific. The glands in *Dionaea* have been particularly well characterized, mainly in terms of enzyme secretion and nutrient absorption (Hedrich and Neher, 2018; Hedrich and Fukushima, 2021). To understand the evolutionary trends of carnivorous plant glands, it is important to study multiple lineages and to apply knowledge about a well-studied species to other species. In addition to studying glands, further research is needed to integrate our fragmentary knowledge about other carnivory-related traits, such as prey attraction and trap development. The convergent evolution of carnivorous plants provides an opportunity to study both common, convergent trends and unique traits in the establishment of glands and other specialized tissues.

ADVANCES

- Glandular structures are common among vascular plants, but many carnivorous plant glands show a distinct, common set of features for digestion and absorption.
- The glands of carnivorous plants secrete mucilage, acids, and proteins, including digestive enzymes, and absorb degraded products using membrane proteins and endocytosis.
- Many genetic components underlying carnivory are tightly linked to defense mechanisms, such as pathogenesis-related proteins and jasmonate-mediated gene regulation.

OUTSTANDING QUESTIONS

- How do the digestive fluids of carnivorous plants achieve the same level of acidity as the gastric juices of some animals?
- Are there convergent evolutionary trends in gland functions among independently evolved carnivorous plants, as well as between carnivorous plants and animals?
- Which cells of noncarnivorous ancestors of a given lineage served as the evolutionary origin of carnivorous glandular cells?
- How were ancestral cellular functions overwritten, repurposed, or reconciled with the new carnivorous functions of glands?
- Which molecular evolutionary mechanisms (e.g. gene duplication with sub-/neofunctionalization and/or new regulatory relationships) led to the convergent co-option of multiple protein families involved in gland functions?

Box 1. Sticky mucilage provides biotic interactions.

Mucilage production has implications for other biotic interactions in carnivorous plants. *Roridula* relies on symbiotic hemipterans living on their traps to digest their prey (Ellis and Midgley, 1996), and similar interactions might also occur in *Byblis* (China and Carvalho, 1951; Hartmeyer, 1998; Lowrie, 1998). A possibly mutualistic, fungivorous mite species was found living in the sticky leaves of *Pinguicula longifolia* (Antor and Garcia, 1995). These symbiotic arthropods require particular biomechanical adaptations to overcome the adhesive forces of these sticky glands and maintain mobility (Voigt and Gorb, 2010). Caterpillars (Fletcher, 1908; Osaki and Tagawa, 2020) and a hoverfly larva (Fleischmann et al., 2016) have also evolved behavioral and physical adaptations to overcome mucilage adhesion to consume the leaves and tentacles or entrapped prey of *Drosera*. Almost nothing is known about the effects of viscoelastic fluid on the aquatic symbionts living in *Nepenthes* pitchers, but one study (Gilbert et al., 2020) revealed little difference in the microbial community composition between species with and without sticky fluid in a greenhouse setting. The nature of the potential microbial and arthropod communities in highly viscoelastic fluid in pitcher plant phytotelmata remains largely unexplored.

Box 2. Transporters enable cation uptake.

Like other plants, carnivorous plants require nitrogen and phosphate, but other elements such as potassium, iron, and manganese are also essential (Adlassnig et al., 2009). The task of potassium uptake in *D.muscipula* is divided between two membrane proteins: the K^+^ transporter 1-like (KT1-like), Shaker-type potassium channel DmKT1, and the high–affinity K^+^ transporter-type (HAK-type) transporter DmHAK5 (Scherzer et al., 2015). The low-affinity, high-capacity channel DmKT1 absorbs the K^+^ released by digestion of prey using the steep K^+^ gradient between the gland cell and the digestive fluid. To avoid the turning point of K^+^-flowback, these channels close in response to low K^+^ concentrations, and the proton-driven transporter DmHAK5 prevents unused K^+^ from being wasted: This transporter has high-potassium affinity but weak selectivity. Sodium absorption is likely to be mediated by the sodium channel DmHKT1, whose transcript level is upregulated by mechanical stimulations and the application of coronatine (Böhm et al., 2016a, 2016b).

## Supplemental data 

The following materials are available in the online version of this article.

**Supplemental Methods S1.** Scanning electron microscopy.

**Supplemental Methods S2.** Methylene blue staining.

**Supplemental Text S1.** Potential roles of the vacuole in gland physiology.

**Supplemental Text S2.** Possible link between large glands and extrafloral nectaries.

**Supplemental Text S3.** Glands of Caryophyllales carnivores.

**Supplemental Text S4.** Glands of Lamiales carnivores.

**Supplemental Text S5.** Glands of Ericales carnivores.

**Supplemental Table S1.** The pH levels of digestive fluids of different species.

**Supplemental References.**

## Supplementary Material

kiac232_Supplementary_Data

## Notes

A.F. to C.S. are ordered alphabetically and V.A.A. and R.H. are ordered alphabetically.

D.G., M.F., and K.F. conceptualized the scope of the manuscript. D.G., M.F., A.F., and Q.L. acquired SEM images. M.F. performed methylene blue staining. D.G., M.F., A.F., Q.L., and K.F. provided plant photographs. C.S. supervised the SEM observation. D.G., M.F., K.J.G., Q.L., A.F., T.R., and K.F. wrote the manuscript with input from all authors. All authors revised and approved the manuscript. K.F. supervised the project. K.F. agrees to serve as the author responsible for contact and ensures communication.

The author responsible for distribution of materials integral to the findings presented in this article in accordance with the policy described in the Instructions for Authors (https://academic.oup.com/plphys/pages/general-instructions) is Kenji Fukushima (kenji.fukushima@uni-wuerzburg.de).
