# Supplementary material for: The digestive systems of carnivorous plants
Source: Plant Physiol. 2022 May 23;190(1):44–59. doi: 10.1093/plphys/kiac232 (PMC9434158; doi:10.1093/plphys/kiac232)
Supplement: kiac232_Supplementary_Data [file kiac232_supplementary_data.zip › PP2022UP00084R1_Supplemental_Materials.pdf]

Supplemental Data

for

The digestive systems of carnivorous plants

Matthias Freund[†], Dorothea Graus[†], Andreas Fleischmann, Kadeem J. Gilbert, Qianshi Lin, Tanya Renner, Christian Stigloher, Victor Albert, Rainer Hedrich, and Kenji Fukushima*

† M.F. and D.G. should be considered joint first authors.

* Correspondence to: K.F.

List of Supplemental Materials:

Supplemental Methods S1–S2
Supplemental Text S1–S5
Supplemental Table S1 (separate file)
Supplemental References
Additional online resources (available from https://doi.org/10.6084/m9.figshare.18271529)

Supplemental Methods

Supplemental Methods S1. Scanning electron microscopy. Samples were fixed overnight in glutaraldehyde solution (6.25% [w/v] glutaraldehyde in 75 mM Sørensen phosphate buffer, pH 7.4) and washed five times with Sørensen phosphate buffer (100 mM, pH 7.4) in 5-minute intervals. Dehydration was performed via a nine-step process using an acetone gradient (30%/50%/75%/90%/5×100%[v/v] acetone for 15/20/30/45/5×30 min). Finally, critical-point-dried (BAL-TEC CPD 030 Critical Point Dryer) samples were sputter-coated in a gold-palladium alloy (BAL-TEC SCD 005 Sputter Coater). Images were taken using a JEOL JSM-7500F field emission scanning electron microscope.

Supplemental Methods S2. Methylene blue staining. Samples were stained with an aqueous 0.1% [w/v] methylene blue solution. *Cephalotus follicularis*, *Sarracenia purpurea*, and *Heliamphora nutans* pitchers were filled with the methylene blue solution. *Darlingtonia californica* received the same treatment, but the hood of the trap was cut open to allow access to the pitcher. *Roridula gorgonias* leaves were removed at the base and completely submerged in the solution. After 10 minutes, the pitchers were removed from the plant and thoroughly rinsed with water to remove excess staining solution. *Roridula* leaves were removed from the bath and rinsed with water as well. Microscopy sections were taken from the digestive zone of the pitcher (lower 15%) and along the midrib of the *Roridula* leaf.

Supplemental Text

Supplemental Text S1. Potential roles of the vacuole in gland physiology. Although its involvement in gland cell physiology is unknown, in non-carnivorous plants, the vacuole builds vesicles that fuse with the plasma membrane (or the vacuole directly fuses with this structure) and release their contents into the apoplast (Echeverría, 2000; Hatsugai et al., 2009; Yun and Kwon, 2017; Shimada et al., 2018). Thus, proteins are safely stored inside the vacuole and are rapidly released in large quantities when needed. In *Dionaea muscipula* gland cells, *AHA10* (encoding a vacuolar proton pump) and *CLC* genes (encoding vacuolar anion transporters) were upregulated in response to coronatine, pointing to a role for the vacuole in digestive physiology (Scherzer et al., 2017).

The vacuole plays a major role in fluid secretion in *Pinguicula* (Heslop-Harrison and Heslop-Harrison, 1981). During the last stage of glandular cell maturation, a controlled type of autolysis takes place, which has been described as interrupted holocrine secretion. During this process, the vacuolar membrane dissolves, and its entire contents are released (Heslop-Harrison and Heslop-Harrison, 1981).

Supplemental Text S2. Possible link between large glands and extrafloral nectaries. *Cephalotus* utilizes extrafloral nectaries to attract its prey (Vogel, 1998; Ellison and Adamec, 2018). The structure of the nectaries resembles that of large glands, but they contain only 25 to 30 cells (Vogel, 1998). The extrafloral nectaries are located on the inner side of the lid, on the teeth of the peristome, and on the outer side of the pitcher wall, whereas most large glands are located in the glandular region inside the trap (Juniper et al., 1989; Vogel, 1998; Ellison and Adamec, 2018).

Supplemental Text S3. Glands of Caryophyllales carnivores. Arguably the most famous and well-studied carnivorous plant is *Dionaea muscipula*, the Venus flytrap. The trapping organ comprises two leaf lobes with teeth-like extensions at their rims, invoking the image of a foothold trap. The inner surfaces of the lobes are covered with digestive glands. The upper, exposed part of each gland is ~100 µm in diameter, comprising secretory cells of a slightly convex shape (Scala et al., 1968). Basal cells anchor the gland to the epidermis and are connected to the secretory cells by a layer of endodermoid cells (Juniper et al., 1989). Upon prey capture, the trap closes and starts releasing digestive fluid through the secretory cells. Additional sessile glands on the trap rim are tasked with the secretion of carbohydrate-rich mucilage (Joel et al., 1985). These glands lie in small indentures, which protect them from physical damage when the trap closes (Juniper et al., 1989).

Aldrovanda vesiculosa is the aquatic sister species to *Dionaea muscipula*. Their traps look very similar, with two lobes connected via a midrib and teeth-like structures sitting at the rim. *Aldrovanda* traps are much smaller (only a few millimeters in size). Digestive glands are densely distributed in the center of the trap. The rim region bears quadrifid glands (sometimes called absorptive hairs (Atsuzawa et al., 2020)): These X-shaped structures closely resemble those of *Utricularia*, another aquatic

carnivore of the distantly related Lentibulariaceae whose quadrifid glands in X-shaped structures likely aid in water removal and digestion (Lloyd, 1942; Fineran and Lee, 1975; Fineran, 1985; Płachno et al., 2007). The idea that *Aldrovanda* glands possess the convergent function of water removal is attractive due to their morphological similarity, but this needs to be confirmed. The glands in *Aldrovanda* likely play a role in digestion, as they show phosphatase activity (Płachno et al., 2006). The teeth-like structures of the snap traps might be modified stalked glands that have lost their secretory function (Heubl et al., 2006).

The genus *Drosera* is a diverse taxon with ca. 260 recognized species (Fleischmann et al., 2018a; Cross et al., 2020). *Drosera* traps come in various shapes and sizes, but they all share common elements. All known *Drosera* species have sticky flypaper traps. The stickiness is provided by stalked glands covering the leaf surface. These glands vary in size, with the longest tentacles sitting at the outer regions and gradually smaller ones toward the center. The glandular head is roughly oval and is usually covered by mucilage droplets, which earned *Drosera* its common name "sundew". When viewed under the microscope, distinct structures inside the glandular head become visible: The outermost cells are epidermal cells with a very thin cuticle (Fenner, 1904; Lloyd, 1942), allowing for easier mucilage excretion. The next layer comprises inner gland cells, which are parenchymal. Below this is a bell-shaped area of endodermoid cells separated by Casparian strips (Fenner, 1904): These lignified cell walls are usually associated with roots, where they function in water transport (Lersten, 1997). Casparian strips are impermeable to water, effectively forcing water through the symplastic pathway (Stöckle and Vermeer, 2020). At the core of the glandular head is the vascular system, a tracheid body, which narrows into a single canal through the stalk (Ragetli et al., 1972). Unlike the flypaper traps of other lineages, the stalked glands move slowly in ca. 90% of the known *Drosera* species (Fleischmann et al., 2018a). Once an insect is stuck on the leaf, tentacles around the insect begin to move towards it, involving more glands to increase prey retention. An exceptionally fast movement occurs in *D. glanduligera*, *D. burmannii*, and some of the Australian pygmy *Drosera* (*D.* section *Bryastrum*), where the outermost tentacles catapult prey directly onto the leaf center (Poppinga et al., 2012). Directly on the leaf surface, among the forest of tentacles, sit much smaller, sessile glands. These glands lack a stalk and typically consist of four head cells, a single-celled neck, and two basal cells. These sessile glands lack vasculature at maturity (Fenner, 1904; Juniper et al., 1989). An additional type of sessile gland with just two head cells has been reported in *D. capensis* (Naidoo and Heneidak, 2013). Sessile glands may aid in digestion by releasing digestive enzymes (Matušíková et al., 2005; Naidoo and Heneidak, 2013), but their role in absorption was put into question by fluorescent tracer experiments (Adlassnig et al., 2012). Their fluid is more liquid than that of stalked glands, providing better solubility of digestive enzymes (Von Byern and Grunwald, 2010).

More distantly related to the Droseraceae, but still part of the Caryophyllales, are two other plants with flypaper traps: *Drosophyllum lusitanicum* and *Triphyophyllum peltatum*. Rather atypical for most carnivorous plants, the monotypic *D. lusitanicum* grows in relatively dry environments (Lloyd,

1942; Adlassnig et al., 2006). Its leaves are elongated and very thin. They are curled up when young and straighten out gradually over time, similar to ferns. On the leaf surface, as well as on the flower pedicle and calyx, are relatively large stalked glands and sessile glands that are visible to the naked eye (Ojeda et al., 2021). The stalked glands are mushroom-shaped and exude mucilage. They do not move in response to prey capture. The sessile glands function in digestion and remain dry until stimulated (Green et al., 1979; Vassilyev, 2005). The stalked and sessile glands of *Drosophyllum* contain xylem and phloem.

Triphyophyllum peltatum is a special case among carnivorous plants in that it is only carnivorous during a short period of its lifetime. While the closely related Ancistrocladaceae lost their carnivory entirely, *T. peltatum* can produce trap leaves when the plant is still young, usually during the rainy season (Green et al., 1979; Fleischmann et al., 2018b). The traps are morphologically similar to those of *Drosophyllum* and grow and uncurl in the same manner. These traps also have both stalked mucilage glands and sessile digestive glands with xylem and phloem (Green et al., 1979; Marburger, 1979). Unlike both *Drosera* and *Drosophyllum*, tracheids branch out extensively inside the stalked glands of *T. peltatum* (Juniper et al., 1989). *Triphyophyllum* glands are the largest among the known flypaper carnivores (and among angiosperms in general, as they are only outsized by the resinous glands of some Velloziaceae), with heads reaching up to 1 mm in diameter (Green et al., 1979).

The last carnivorous group of the Caryophyllales (regarding trap type) is the Nepenthaceae. *Nepenthes* species typically produce large, elaborate pitcher traps. A short, flexuous tendril sprouts off its flattened, photosynthetic petiole, which serves as the connection between the pitcher proper and the rest of the plant. The pitchers themselves are often funnel-shaped, as they are more bulbous at the bottom (depending on the species) and open up toward the top. The entrance to the pitcher is marked by a thick collar—the peristome—with short tooth-like protrusions and a leaf hanging above as a lid. Nectary glands in small cavities between the teeth attract prey (Joel, 1988; Moran, 1996; Merbach et al., 2001). At the very bottom of the *Nepenthes* pitcher is the digestive zone. Here, large digestive glands maintain the digestive fluid by releasing enzymes and absorbing nutrients (An et al., 2002; Adlassnig et al., 2012). Cells in the topmost layer of the digestive glands have thick walls and a thin cuticle facing the inside of the pitcher. A continuous layer of epidermal cells curves underneath the gland, with vascular cells in close proximity. A piece of epidermis arches above each digestive gland (Owen, Jr., 1999; Wang et al., 2009); these lunate ridges resemble stomatal cells. More lunate cells can be found near the peristome, albeit without glands beneath them (Wang et al., 2018). Due to their morphology, lunate cells are thought to originate from guard cells and to interfere with insect movement by providing a difficult surface in conjunction with particular wax crystals (Wang et al., 2009; Wang et al., 2016; Wang et al., 2018).

The flowers of *Plumbago* (Plumbaginaceae), a non-carnivorous taxon from the sister group to the carnivorous lineage in Caryophyllales, bear sticky glandular trichomes, superficially resembling those of *Drosophyllum*, but with little or no vascularization (Stoltzfus et al., 2002). Although these

trichomes can trap insects and secrete digestive enzymes, the plant does not appear to make use of the digested nutrients (Stoltzfus et al., 2002). Unlike in *Triantha*, whose glands on flower stalks are utilized for carnivory (Lin et al., 2021), the glandular trichomes on floral organs of other plants are thought to function in defense against herbivory (Kerner von Marilaun, 1878). The flower is an expensive and critical organ for plants, making it advantageous to invest in its protection ("optimal defense theory") (McKey, 1974; McKey, 1979; Rhoades, 1979).

Supplemental Text S4. **Glands of Lamiales carnivores.** The sticky leaves of ca. 110 species of *Pinguicula* are usually arranged in a rosette, with each leaf heavily covered in stalked and sessile glands. Below each stalked gland sits a basal reservoir cell, which is connected to four to eight surrounding cells via plasmodesmata (Heslop-Harrison and Heslop-Harrison, 1981). The stalks of these glandular trichomes are single-celled and shaped like a bowling pin: thicker near the base and narrowing towards the head. The head itself is composed of a single cell surrounded by 16 roughly symmetrically distributed cells (Fenner, 1904; Juniper et al., 1989). The sessile glands are arranged in a similar manner, but without the stalk and surrounded by just eight cells (Fenner, 1904; Lloyd, 1942; Heslop-Harrison and Heslop-Harrison, 1981). Underneath the secretory head of the sessile gland is a single endodermoid cell, followed by another single reservoir cell (Heslop-Harrison and Heslop-Harrison, 1981).

Genlisea is a unique genus. Instead of roots, its 30 species bear subterranean leaves (Fleischmann, 2012; Fleischmann, 2018). These leaves lack chlorophyll and form a tube, which branches off into two "arms". Each arm is twisted like a corkscrew, providing an entrance for potential prey into the trap. Inward-pointing trichomes allow the prey to only move further inside. The prey eventually ends up inside a thicker part of the tube: a digestive chamber. On the inner surface of the digestive chamber are digestive glands, which are thought to continuously release digestive fluid into the chamber (Płachno et al., 2007). An ancestor to *Genlisea* might have actively transported water (Jobson et al., 2004), and the positioning of digestive glands could be a remnant of this (Płachno et al., 2007; Fleischmann, 2012). The glands are made of three types of cells: terminal cells in radial arrangement, one endodermoid cell, and one basal cell (Lloyd, 1942; Płachno et al., 2007). These cells take on both secretory and absorptive functions (Fleischmann, 2012).

Utricularia is an immediate sister genus to *Genlisea* containing both terrestrial and aquatic species. These species lack roots, instead bearing subterranean or aquatic trap leaves. These traps form a bladder whose entrance contains sensory trigger hairs and is sealed off by a trap door (Lloyd, 1942; Reifenrath et al., 2006; Westermeier et al., 2017). To arm the trap, water inside must be evacuated to reduce the water pressure inside (Lloyd, 1942). When the prey disturbs the trigger hairs at the trap door entrance, it becomes sucked inside the trap via the rapid bulging of bladder walls (Lloyd, 1942). The trap door is covered with stalked mucilage glands, which are thought to seal the trap door following prey capture (Westermeier et al., 2017; Płachno et al., 2019). Along the inside of the trap walls are bifid or quadrifid glands. Their terminal cells exhibit a highly complex cell wall labyrinth, which might

enable vigorous water transport (Fineran and Lee, 1975; Sasago and Sibaoka, 1985; Płachno et al., 2007). *Utricularia* might use these glands to rapidly remove water from the trap (Lloyd, 1942; Fineran and Lee, 1980; Fineran, 1985) and for absorptive and secretory processes (Lloyd, 1942; Fineran and Lee, 1975; Fineran, 1985; Sirová et al., 2003; Juang et al., 2011).

In *Byblis*, like most species of *Pinguicula*, most of the aerial parts of the plant are densely covered in mucilage glands, up to and including the floral scapes and sepals (Juniper et al., 1989; Kocáb et al., 2020). Its stalked glands are highly similar to those of *Pinguicula* as well, with a single-celled stalk and a radial arrangement of head cells (Fenner, 1904; Juniper et al., 1989). However, rather than sitting on top of a singular basal cell, *Byblis* stalked glands are surrounded by smaller epidermal cells (Fenner, 1904; Juniper et al., 1989).

A relatively recent addition to the cadre of lamialean carnivorous plants is *Philcoxia*. This genus currently comprises seven species endemic to Brazil (Scatigna et al., 2018). *Philcoxia* plants have minute, green leaves, which are usually buried beneath a thin layer of sand and prey upon nematodes with their sticky traps (Pereira et al., 2012). While early studies could not confirm phosphatase activity in the glandular trichomes of these plants, more recent reports of phosphatase activity in *P. minensis* and the presence of nematodes stuck on the leaves of *P. rhizomatosa* categorize *Philcoxia* as a carnivorous plant (Pereira et al., 2012; Scatigna et al., 2015; Scatigna et al., 2017; Fleischmann et al., 2018b). In addition to stalked glands that secrete mucilage, sessile glands have been described (Taylor et al., 2000), although whether they are universally present in this genus is not clear.

Supplemental Text S5. **Glands of Ericales carnivores.** There is little evidence for the presence of digestive enzymes in *Roridula* glands and their resinous exudate (Lloyd, 1934). Płachno et al. studied phosphatase activity in different carnivorous lineages and did not observe fluorescence in *Roridula* stalked glands, but rather in the epidermis of the leaf surface (Płachno et al., 2006), which constitutes a physiologically highly active "digestive epithelium" for nutrient uptake from the feces of associated mutualistic arthropods (Anderson, 2005). A follow-up study found that despite the absence of phosphatase activity in the glands (and the absence of a symbiotic insect), phosphate uptake was comparable to that of *Drosophyllum lusitanicum* (Płachno et al., 2009). There may be an evolutionary connection between this phenomenon and digestion in the Sarraceniaceae. Both Roridulaceae and Sarraceniaceae appear to absorb nutrients using relatively simple glands comprising specialized epidermal cells bearing thin cuticles for easier transport, whereas other carnivorous lineages utilize more complex structures for the same task.

One hypothesis on how carnivory in plants could evolve is via "foliar feeding" (Fernández and Eichert, 2009; Fernández and Brown, 2013) on hairy, non-carnivorous leaves. Dense trichomes retain sticky droplets that catch insect parts and other organic matter. The released nutrients could then be absorbed through the epidermis. Under this scenario, carnivory in the Ericales would represent a step between foliar feeding and advanced carnivory. The extrafloral glands of Ericales secrete resin, not

aqueous mucilage, but enzymes cannot dissolve prey in lipophilic resin; this may explain the origin of digestive mutualism with capsid bug partners in *Roridula* (Anderson, 2005; Fleischmann et al., 2018b).

Supplemental Table

Supplemental Table S1. The pH levels of digestive fluids of different species**.** (separate file)

Supplemental References

Adlassnig W, Koller-Peroutka M, Bauer S, Koshkin E, Lendl T, Lichtscheidl IK (2012) Endocytotic uptake of nutrients in carnivorous plants. Plant J **71**: 303–313

Adlassnig W, Peroutka M, Eder G, Pois W, Lichtscheidl IK (2006) Ecophysiological observations on *Drosophyllum lusitanicum*. Ecol Res **21**: 255–262

An C-I, Takekawa S, Okazawa A, Fukusaki E, Kobayashi A (2002) Degradation of a peptide in pitcher fluid of the carnivorous plant *Nepenthes alata* Blanco. Planta **215**: 472–477

Anderson B (2005) Adaptations to foliar absorption of faeces: A pathway in plant carnivory. Ann Bot **95**: 757–761

Atsuzawa K, Kanaizumi D, Ajisaka M, Kamada T, Sakamoto K, Matsushima H, Kaneko Y (2020) Fine structure of *Aldrovanda vesiculosa* L: the peculiar lifestyle of an aquatic carnivorous plant elucidated by electron microscopy using cryo-techniques. Microscopy **69**: 214–226

Cross AT, Krueger TA, Gonella PM, Robinson AS, Fleischmann AS (2020) Conservation of carnivorous plants in the age of extinction. Glob Ecol Conserv **24**: e01272

Echeverría E (2000) Vesicle-mediated solute transport between the vacuole and the plasma membrane. Plant Physiol **123**: 1217–1226

Ellison AM, Adamec L (2018) Carnivorous plants: physiology, ecology, and evolution. Oxford University Press

Fenner CA (1904) Beiträge zur Kenntnis der Anatomie, Entwicklungsgeschichte und Biologie der Laubblätter und Drüsen einiger Insektivoren. Val. Höfling

Fernández V, Brown PH (2013) From plant surface to plant metabolism: the uncertain fate of foliar-applied nutrients. Front Plant Sci. doi: 10.3389/fpls.2013.00289

Fernández V, Eichert T (2009) Uptake of hydrophilic solutes through plant leaves: Current state of knowledge and perspectives of foliar fertilization. Crit Rev Plant Sci **28**: 36–68

Fineran BA (1985) Glandular trichomes in *Utricularia*: a review of their structure and function. 38

Fineran BA, Lee MSL (1975) Organization of quadrifid and bifid hairs in the trap of *Utricularia monanthos*. Protoplasma **84**: 43–70

Fineran BA, Lee MSL (1980) Organization of mature external glands on the trap and other organs of the bladderwort *Utricularia monanthos*. Protoplasma **103**: 17–34

Fleischmann A (2012) Monograph of the genus *Genlisea*. Redfern Natural History Productions, Poole, Dorset

Fleischmann A (2018) Systematics and evolution of Lentibulariaceae: II. *Genlisea*. Carniv. Plants Physiol. Ecol. Evol. Oxford University Press, Oxford, pp 81–88

Fleischmann A, Cross AT, Gibson R, Gonella PM, Dixon KW (2018a) Systematics and evolution of Droseraceae. Carniv. Plants Physiol. Ecol. Evol. Part II Syst. Evol. Carniv. Plants. Oxford University Press, Oxford, pp 45–57

Fleischmann A, Schlauer J, Smith SA, Givnish TJ (2018b) Evolution of carnivory in angiosperms. doi: 10.1093/oso/9780198779841.003.0003

Green S, Green TL, Heslop-Harrison Y (1979) Seasonal heterophylly and leaf gland features in *Triphyophyllum* (Dioncophyllaceae), a new carnivorous plant genus. Bot J Linn Soc **78**: 99–116

Hatsugai N, Iwasaki S, Tamura K, Kondo M, Fuji K, Ogasawara K, Nishimura M, Hara-Nishimura I (2009) A novel membrane fusion-mediated plant immunity against bacterial pathogens. Genes Dev **23**: 2496–2506

Heslop-Harrison Y, Heslop-Harrison J (1981) The digestive glands of *Pinguicula*: Structure and cytochemistry. Ann Bot **47**: 293–319

Heubl G, Bringmann G, Meimberg H (2006) Molecular phylogeny and character evolution of carnivorous plant families in caryophyllales — revisited. Plant Biol **8**: 821–830

Jobson RW, Nielsen R, Laakkonen L, Wikstrom M, Albert VA (2004) Adaptive evolution of cytochrome c oxidase: Infrastructure for a carnivorous plant radiation. Proc Natl Acad Sci **101**: 18064–18068

Joel DM (1988) Mimicry and mutualism in carnivorous pitcher plants (Sarraceniaceae, Nepenthaceae, Cephalotaceae, Bromeliaceae). Biol J Linn Soc **35**: 185–197

Joel DM, Juniper BE, Dafni A (1985) Ultraviolet patterns in the traps of carnivorous plants. New Phytol **101**: 585–593

Juang TC-C, Juang SD-C, Liu Z-H (2011) Direct evidence of the symplastic pathway in the trap of the bladderwort *Utricularia gibba L.* Bot Stud **52**: 47–54

Juniper BE, Robins RJ, Joel DM (1989) The Carnivorous Plants. Academic Press, London

Kerner von Marilaun A (1878) Flowers and their unbidden guests. C.K. Paul & co., London

Kocáb O, Jakšová J, Novák O, Petřík I, Lenobel R, Chamrád I, Pavlovič A (2020) Jasmonate-independent regulation of digestive enzyme activity in the carnivorous butterwort *Pinguicula* × *Tina*. J Exp Bot **71**: 3749–3758

Lersten NR (1997) Occurrence of endodermis with a casparian strip in stem and leaf. Bot Rev **63**: 265–272

Lin Q, Ané C, Givnish TJ, Graham SW (2021) A new carnivorous plant lineage (*Triantha*) with a unique sticky-inflorescence trap. Proc Natl Acad Sci **118**: e2022724118

Lloyd FE (1942) The carnivorous plants. Chronica Botanica Company, Waltham, WA, USA

Lloyd FE (1934) Is *Roridula* a carnivorous plant? Can J Res **10**: 780–786

Marburger JE (1979) Glandular leaf structure of *Triphyophyllum peltatum* (Dioncophyllaceae): A "fly-paper" insect trapper. Am J Bot **66**: 404–411

Matušíková I, Salaj J, Moravčíková J, Mlynárová L, Nap J-P, Libantová J (2005) Tentacles of in vitro-grown round-leaf sundew (*Drosera rotundifolia* L.) show induction of chitinase activity upon mimicking the presence of prey. Planta **222**: 1020–1027

McKey D (1974) Adaptive patterns in alkaloid physiology. Am Midl Nat **108**: 305–320

McKey D (1979) The distribution of secondary compounds within plants. G Rosenthal H Janzen Eds Herbiv. Their Interact. Second. Plant Metab. Academic Press, Orlando, Fla, pp 56–134

Merbach MA, Zizka G, Fiala B, Maschwitz U, Booth WE (2001) Patterns of nectar secretion in five *Nepenthes* species from Brunei Darussalam, Northwest Borneo, and implications for ant-plant relationships. Flora **196**: 153–160

Moran JA (1996) Pitcher dimorphism, prey composition and the mechanisms of prey attraction in the pitcher plant *Nepenthes rafflesiana* in Borneo. J Ecol **84**: 515

Naidoo Y, Heneidak S (2013) Morphological investigation of glandular hairs on *Drosera capensis* leaves with an ultrastructural study of the sessile glands. Botany **91**: 234–241

Ojeda F, Carrera C, Paniw M, García-Moreno L, Barbero GF, Palma M (2021) Volatile and semi-volatile organic compounds may help reduce pollinator-prey overlap in the carnivorous plant *Drosophyllum lusitanicum* (Drosophyllaceae). J Chem Ecol **47**: 73–86

Owen, Jr. T (1999) Pathways for nutrient transport in the pitchers of the carnivorous plant *Nepenthes alata*. Ann Bot **84**: 459–466

Pereira CG, Almenara DP, Winter CE, Fritsch PW, Lambers H, Oliveira RS (2012) Underground leaves of *Philcoxia* trap and digest nematodes. Proc Natl Acad Sci U S A **109**: 1154–1158

Płachno BJ, Adamec L, Huet H (2009) Mineral nutrient uptake from prey and glandular phosphatase activity as a dual test of carnivory in semi-desert plants with glandular leaves suspected of carnivory. Ann Bot **104**: 649–654

Płachno BJ, Adamec L, Lichtscheidl IK, Peroutka M, Adlassnig W, Vrba J (2006) Fluorescence labelling of phosphatase activity in digestive glands of carnivorous plants. Plant Biol **8**: 813–820

Płachno BJ, Kozieradzka-Kiszkurno M, Świątek P (2007) Functional ultrastructure of *Genlisea* (Lentibulariaceae) digestive hairs. Ann Bot **100**: 195–203

Płachno BJ, Świątek P, Miranda VFO, Stolarczyk P (2019) The structure and occurrence of a velum in *Utricularia* traps (Lentibulariaceae). Front Plant Sci **10**: 302

Poppinga S, Hartmeyer SRH, Seidel R, Masselter T, Hartmeyer I, Speck T (2012) Catapulting tentacles in a sticky carnivorous plant. PLOS ONE **7**: e45735

Ragetli HWJ, Weintraub M, Lo E (1972) Characteristics of *Drosera* tentacles. I. Anatomical and cytological detail. Can J Bot **50**: 159–168

Reifenrath K, Theisen I, Schnitzler J, Porembski S, Barthlott W (2006) Trap architecture in carnivorous *Utricularia* (Lentibulariaceae). Flora - Morphol Distrib Funct Ecol Plants **201**: 597–605

Rhoades D (1979) Evolution of plant chemical defense against herbivores. G Rosenthal H Janzen Eds

Herbiv. Their Interact. Second. Plant Metab. Academic Press, New York, pp 4–55
Sasago A, Sibaoka T (1985) Water extrusion in the trap bladders of *Utricularia vulgaris* I. A possible pathway of water across the bladder wall. Bot Mag Tokyo **98**: 55–66
Scala J, Schwab D, Simmons E (1968) The fine structure of the digestive gland of Venus's Flytrap. Am J Bot **55**: 649–657
Scatigna AV, Fritsch PW, Souza VC, Simões AO (2018) Phylogenetic relationships and morphological evolution in the carnivorous genus *Philcoxia* (Plantaginaceae, Gratioleae). Syst Bot **43**: 910–919
Scatigna AV, Souza VC, Pereira CG, Sartori MA, Simões AO (2015) *Philcoxia rhizomatosa* (Gratioleae, Plantaginaceae): a new carnivorous species from Minas Gerais, Brazil. Phytotaxa **226**: 275
Scatigna AV, Souza VC, Simões AO (2017) *Stemodia cipoensis* (Plantaginaceae): A new species from Serra do Cipó, Minas Gerais, Brazil. Syst Bot **42**: 371–377
Scherzer S, Shabala L, Hedrich B, Fromm J, Bauer H, Munz E, Jakob P, Al-Rascheid KAS, Kreuzer I, Becker D, et al (2017) Insect haptoelectrical stimulation of Venus flytrap triggers exocytosis in gland cells. Proc Natl Acad Sci U S A **114**: 4822–4827
Shimada T, Takagi J, Ichino T, Shirakawa M, Hara-Nishimura I (2018) Plant Vacuoles. Annu Rev Plant Biol **69**: 123–145
Sirová D, Adamec L, Vrba J (2003) Enzymatic activities in traps of four aquatic species of the carnivorous genus *Utricularia*. New Phytol **159**: 669–675
Stöckle D, Vermeer JEM (2020) Plant biology: Journey to the center of the casparian strip. Curr Biol **30**: R1273–R1275
Stoltzfus A, Suda J, Kettering A, Wolfe A, Willia S (2002) Secretion of digestive enzymes in *Plumbago*. Phylogeny Carniv. Plants. Tokyo, pp 203–207
Taylor P, Souza VC, Giulietti AM, Harley RM (2000) *Philcoxia*: a new genus of Scrophulariaceae with three new species from eastern Brazil. Kew Bull **55**: 155–163
Vassilyev AE (2005) Dynamics of ultrastructural characters of *Drosophyllum lusitanicum* link (Droseraceae) digestive glands during maturation and after stimulation. Taiwania **50**: 167–182
Vogel S (1998) Remarkable nectaries: structure, ecology, organophyletic perspectives. Flora **193**: 1–29
Von Byern J, Grunwald I, eds (2010) Biological adhesion systems: from nature to technical and medical application. Springer, Vienna
Wang L, Dong S, Zhou Q (2016) Slippery Surface of *Nepenthes alata* Pitcher: The role of lunate cell and wax crystal in restricting attachment ability of ant *Camponotus japonicus* Mayr. J Bionic Eng **13**: 373–387
Wang L, Tao D, Dong S, Li S, Tian Y (2018) Contributions of lunate cells and wax crystals to the surface anisotropy of *Nepenthes* slippery zone. R Soc Open Sci **5**: 180766
Wang L, Zhou Q, Zheng Y, Xu S (2009) Composite structure and properties of the pitcher surface of the carnivorous plant *Nepenthes* and its influence on the insect attachment system. Prog Nat Sci **19**: 1657–1664
Westermeier AS, Fleischmann A, Müller K, Schäferhoff B, Rubach C, Speck T, Poppinga S (2017) Trap diversity and character evolution in carnivorous bladderworts (*Utricularia*, Lentibulariaceae). Sci Rep **7**: 12052
Yun HS, Kwon C (2017) Vesicle trafficking in plant immunity. Curr Opin Plant Biol **40**: 34–42
